# Health system preparedness for newborn care: a health facility assessment in rural Uganda

**DOI:** 10.1186/1472-6963-14-S2-P63

**Published:** 2014-07-07

**Authors:** Christine Kayemba Nalwadda, Stefan Peterson, Goran Tomson, David Guwatudde, Juliet Kiguli, Gertrude Namazzi, Sarah Namutamba, Faith Namugaya, Harriet Nambuya, Abner Tagoola, Peter Waiswa

**Affiliations:** 1 School of Public Health, Makerere University, Kampala, Uganda; 2 Health System and Policy, Department of Public Health Sciences, Karolinska Institutet, Stockholm, Sweden; 3 International Maternal and Child Health Unit, Department of Women and Children Health, Uppsala University, Uppsala, Sweden; 4 Uganda Newborn Survival Study, Iganga-Mayuge Health Demographic Surveillance Site, Kampala, Uganda

## Background

Newborn deaths must be reduced to achieve Millennium Development Goal four. Health facilities have a critical role to play in the fight to save the 2.9 million newborns that die in the world every year. It is not clear if health facilities in rural Uganda have the capacity to care for newborns.

## Objective

To assess the capacity of health facilities to care for newborns in Iganga and Mayuge districts in eastern Uganda for the three main mortality causes: preterm/low birth weight, asphyxia and sepsis.

## Materials and methods

Between July and August 2013, a cross-sectional study was conducted among 92 health workers, and in 20 health facilities: one hospital and 19 primary health care centres in areas where some health facility strengthening for newborn care had occurred. The indicators measured included; services offered, equipment, drugs and supplies, documentation, trained staff and supervision, health worker knowledge and resuscitation skills for newborns. STATA version 10 was used to analyze the data and availability scores were generated by using the Service Availability and Readiness Assessment, a World Health Organisation methodology for measuring health systems strengthening.

## Results

Fifteen of the 20 health facilities offered newborn care. First level facilities (Level II**)** had the lowest (31%) availability score for resuscitation equipment compared to the hospital/level IV (71%) and those at level III (74%). None of the level II facilities offered kangaroo mother care services for preterm/ low birth weight, while the availability score for this service was 67% for level III and 100% for the hospital/ level IV. Availability score for newborn sepsis drugs was 8% for level II, (67%) and (75%) for level III and the hospital/level IV respectively. Over two thirds (33/50, 66%) of the health workers were considered knowledgeable in newborn care, but less than a half (17/42, 41%) skilled in newborn resuscitation.

## Conclusions

Health workers had good knowledge but modest skills for newborn care. Overall, higher level health facilities were more prepared for newborn care than the first level ones. All first level facilities should be enabled by policy and in practice to treat newborn sepsis. All health facilities that conduct deliveries irrespective of the level of service should also provide good quality preterm/ low birth weight and asphyxia care.

